# Sebaceous Carcinoma of the Nose: A Rare Presentation of an Uncommon Tumor

**Published:** 2019-03-27

**Authors:** Katherine Braunlich, Brian Wanner, Richard Miller

**Affiliations:** Department of Dermatology, Largo Medical Center, Nova Southeastern University, Largo, Fla

**Keywords:** adipophilin, epithelial membrane antigen, nose, radiation, sebaceous carcinoma

## CASE DESCRIPTION

**Figure 1:**
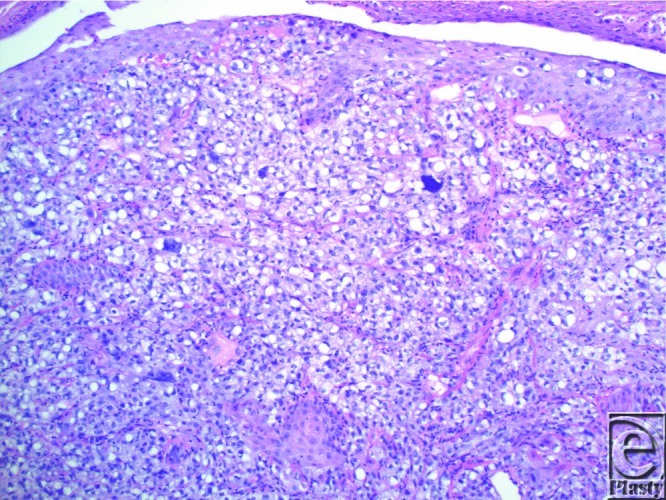
Hematoxylin and eosin revealed an asymmetric, infiltrating aggregation of mature (vacuolated) and immature (nonvacuolated) neoplastic sebocytes.

**Figure 2:**
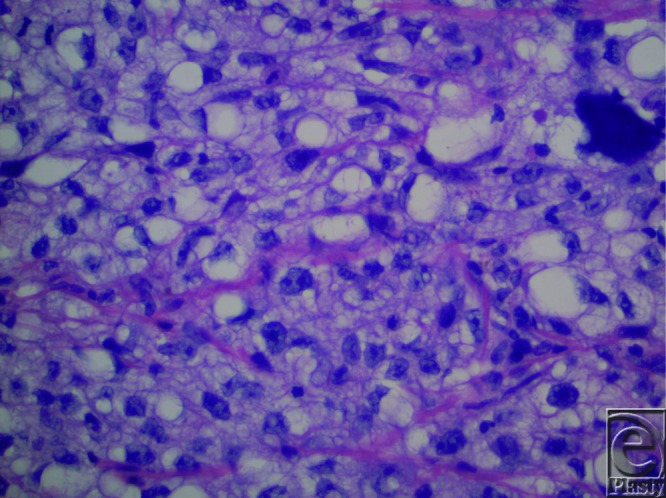
High-power examination, hematoxylin and eosin, revealed prominent nucleoli, pleomorphism, hyperchromatic irregular nuclei, and mitoses.

**Figure 3:**
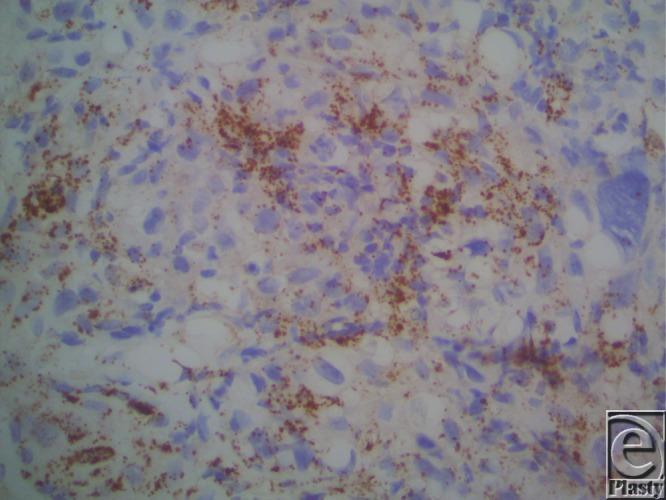
Immunohistochemistry revealed positive staining with adipophilin.

**Figure 4:**
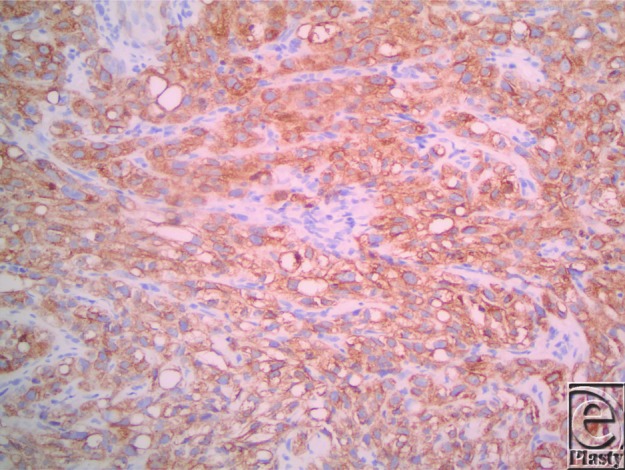
Immunohistochemistry revealed positive staining with epithelial membrane antigen.

We present a case of sebaceous carcinoma (SC) in an uncommon location, the nose. Our patient was a 71-year-old, retired, white male who presented with a 3-month history of a nonhealing lesion on his right nostril. His past medical history was positive for Crohn disease treated with 6-mercaptopurine for 4 years, hypertension (taking atenolol and lisinopril), and peptic ulcer disease. Physical examination revealed a 2.0-cm erosion on the floor of the nostril with extension to the free margin of the ala and columella. Shave biopsy was preformed, and histopathological examination revealed an irritated and inflamed squamous cell carcinoma (SCC). All treatment options were discussed, and the patient elected to proceed with radiation. The patient underwent 33 fractions of radiotherapy over a span of 3 months. Two months following radiation, the patient developed pain and erythema of the nasal tip. Culture and sensitivity returned heavy growth of *Pseudomonas aeruginosa* and *Klebsiella oxytoca*. He was diagnosed with soft tissue cellulitis and treated with ciprofloxacin, mupirocin ointment, and ketorolac. Two weeks later, he developed central facial erythema and ulceration of the right nasal cavity. Shave biopsy revealed an asymmetric, infiltrating aggregation of mature and immature neoplastic sebocytes with pleomorphism, hyperchromatic irregular nuclei, and mitoses (Figs 1 and 2). Immunohistochemistry revealed positive adipophilin and epithelial membrane antigen (EMA) (Figs 3 and 4, respectively). The patient was diagnosed with poorly differentiated SC. The patient underwent radical excision of a 2.4 × 1.8 × 1.2-cm poorly differentiated SC with lymphovascular and perineural invasion. The patient remains in the care of a maxillofacial prosthodontist and a plastic surgeon for osseointegrated implants and nasal prosthesis.

## QUESTIONS

1. What anatomical location accounts for 75% of all SCs?
2. What rare hereditary syndrome is associated with multiple sebaceous neoplasms?
3. What is the most common internal malignancy associated with Muir-Torre syndrome?
4. What is the mainstay of treatment of SC?

## DISCUSSION

Sebaceous carcinoma is a rare, slow-growing, aggressive neoplasm. Seventy-five percent of SCs originate from sebaceous glands in the periocular region, and the remaining are extraocular.^1^^-^8 Extraocular SCs have a higher all-cause mortality than periocular SCs and occur most commonly on the head and neck.^2^^,^^3^^,^^7^ Because of the rarity of extraocular SCs, there have been few reported cases, particularly of nasal SCs.^1^^,^^5^^,^^6^ Our interesting case presents a patient with poorly differentiated nasal SC diagnosed after receiving years of immunosuppressive therapy and recent radiation at the same location for biopsy-proven SCC.

While radiation may be a risk factor for SCs, the precise etiology remains unknown. Recent research suggests that impaired mismatch repair (MMR) proteins might play a role.^7^ This is due to the association of sebaceous neoplasms with Muir-Torre syndrome: an autosomal dominant genodermatosis characterized by sebaceous neoplasms, keratoacanthomas, and internal malignancy.

The most common internal malignancy associated with Muir-Torre syndrome is colorectal carcinoma. Muir-Torre syndrome is caused by mutations in MMR genes, most commonly *MSH2* and *MLH1*, resulting in microsatellite instability (MSI) within DNA.^1^^-^8 Since MSI in Muir-Torre syndrome can potentiate tumor growth and result in SCs, sporadic SCs may be due to the same process. Recent literature suggests that immunosuppression may unveil Muir-Torre syndrome in genetically susceptible individuals and cause oncogenesis.^7^ It is possible that our patient's history of immunosuppression with 6-mercaptopurine played a role in the development of his SC.

Recently, immunohistochemistry has played an important role in aiding in the diagnosis of SC. Using this method, cells with sebaceous differentiation will stain positively for adipophilin, EMA (Figs C and D, respectively), cytokeratin (CAM 5.2), Ber-EP4, and androgen receptor.^7^^,^^8^ Once diagnosed, the mainstay of treatment is wide local excision. This case demonstrates the importance of considering SC for any lesion on the nose, particularly in patients with a history of immunosuppression and/or radiation therapy.

## SUMMARY

Although SC of the nose has been reported previously, it is diagnosed infrequently. The unusual presentation and history of our patient make this case unique.
